# The Effect of Preinduction Cervical Ripening With Synthetic Hygroscopic Dilators on Maternal Outcomes of Women With Previous Caesarean Pregnancy: A Single-Group Clinical Trial

**DOI:** 10.1155/jp/8835464

**Published:** 2024-12-19

**Authors:** Gowri Dorairajan, Saranya Ravi, Palanivel Chinnakili

**Affiliations:** ^1^Department of Obstetrics and Gynaecology, Jawaharlal Institute of Postgraduate Medical Education and Research (JIPMER), Puducherry, India; ^2^Department of Preventive and Social Medicine, Jawaharlal Institute of Postgraduate Medical Education and Research (JIPMER), Puducherry, India

## Abstract

**Background:** Preinduction cervical ripening in previous caesarean pregnancy is limited to intracervical Foley catheter. This study is aimed at finding the vaginal birth rates, improvement of Bishop score, and safety of osmotic dilator (Dilapan-S) among women with previous caesarean pregnancy.

**Methods:** We conducted this single-group clinical study after the approval of the institute ethics committee, clinical trial registration, and obtaining informed consent. We recruited women above 18 years with a prior caesarean section at term and a Bishop score of less than 6 by systematic random sampling prospectively. The first or second author inserted two to a maximum of five osmotic dilators (Dilapan-S) in the cervical canal. After 24 h, we removed Dilapan and induced labour with a low-dose oxytocin regimen up to a maximum dose of 24 mIU/min. We assessed the improvement of the Bishop score and vaginal birth rates for efficacy and safety concerns like bleeding, fragmentation, displacement, infections, and scar dehiscence.

**Results:** Eighty-two women completed the study. The Bishop score significantly improved from a mean of 2.6 before to 5.3 after Dilapan. Three opted for a caesarean section after Dilapan removal and refused oxytocin infusion. Seventy-nine women completed the trial of labour. Forty-one (52%) achieved active labour (52%). Twenty-seven delivered vaginally, and 52 required emergency caesarean section (34% vaginal birth rate; 18 spontaneous, nine instrumental, four with forceps, and five with vacuum). None had entrapment, fragmentation, or upward displacement of Dilapan. Two women had scar dehiscence, and one had a traumatic postpartum haemorrhage. There was no maternal or perinatal mortality.

**Conclusions:** We conclude that the hygroscopic dilator Dilapan effectively ripens the cervix before labour induction in women with a previous caesarean scar. They are safe, but more extensive studies are needed to evaluate scar-related complications during labour.

**Trial Registration**: Clinical Trial Registry of India: CTRI/2019/03/017927

## 1. Introduction

### 1.1. Background

Trial of labour in women with a previous caesarean section (TOLAC) is recommended by the Royal College of Obstetricians and Gynaecologists (RCOG) [1] as well as the American College of Obstetricians and Gynecologists (ACOG) [2]. Induction of labour is a procedure frequently carried out to achieve childbirth when the risk of continuing pregnancy due to maternal or fetal complications outweighs the benefit of continuing pregnancy.

A mature or ripe cervix (Bishop score > 6) is an essential prerequisite for induction of labour to be successful. Mechanical methods like a cervical ripening balloon/Foley catheter balloon inserted in the cervix are the only available options in women with previous caesarean sections. Prostaglandins like misoprostol are contraindicated in women with previous uterine scars [1], and prostaglandin E2 is used with caution. The successful vaginal delivery rate with a Foley balloon inserted in the cervix in women with a scarred uterus is around 50%–60% [3, 4]. Clinicians have been using hygroscopic dilators for midtrimester abortions for many years. Osmotic or hygroscopic dilators are synthetic small rods containing Aquacryl hydrogel. They act by absorbing fluid from the endocervical canal. They swell up and cause dilation of the cervix [5]. Researchers have recently studied the use of synthetic hygroscopic dilator, Dilapan, for induction of labour at term pregnancy and found it to be effective and safe. Most of the studies excluded previous caesarean pregnancies. We undertook this study to determine vaginal birth rates and the safety of osmotic dilator Dilapan-S among women with prior caesarean sections with unfavourable cervix undergoing induction of labour at term.

## 2. Materials and Methods

### 2.1. Study Design, Setting, and Population

We conducted this single-group clinical trial in the Women and Child Block of Jawaharlal Institute of Postgraduate Medical Education and Research. The institute is a tertiary care teaching hospital in Puducherry, India. We conducted the study after the institute ethics committee's approval (JIP/IEC/2018/422 dated Dec 31, 2018) and after prospective registration with the Clinical Trial Registry of India (date of registration: Mar 6, 2019; date of first enrolment: Mar 13, 2019). Once we completed the recruitment of the participants based on the sample size, the study was closed for enrollment in December 2022. We enrolled women fulfilling the inclusion criteria after obtaining informed consent.

### 2.2. Inclusion and Exclusion Criteria

We included women over 18 years with a previous lower segment caesarean section and a singleton fetus in vertex presentation with a Bishop score less than 6, eligible and willing to participate in a trial, requiring labour induction. Besides the RCOG guidelines for the eligibility of trial labour after caesarean, we included only those with a scar thickness ≥ 3 mm and estimated weight below 3500 g.

We induced women without any other obstetric risk factors at 40 weeks of pregnancy for induction of labour. A previous study reported higher vaginal birth rates when such women are induced at 40 weeks instead of expectant management [6]. We excluded women with premature rupture of membranes. We planned systematic random sampling (every third woman eligible for the study). The eligibility criteria remained the same after the trial commenced. The first and the second authors enrolled the participants. There was no blinding after assignment to intervention or outcome measurement.

### 2.3. Sample Size

The successful TOLAC rates have increased over the last decade to up to 75% [7]. Presuming we can achieve a vaginal birth rate of 70% with osmotic dilators for a 5% alpha error (95% confidence level) and absolute precision of 10%, we needed 81 eligible women for the study (OpenEpi version 3).

### 2.4. Intervention Details With Standardization

The second author noted the preinduction Bishop score and other sociodemographic and obstetric details of the 82 participants recruited. None of the women were in the latent phase of labour. In the lithotomy position, under all aseptic precautions, we exposed the cervix with a speculum and held it with sponge-holding forceps. Two osmotic dilators (Dilapan-S) (manufactured by MEDICEM Technology s.r.o., Czech Republic) up to a maximum of five (depending on the caliber of the internal os to accommodate at the time of insertion) were inserted at the same time by the first or second author into the cervical canal and kept for 24 h. All the study participants remained hospitalized. The women were monitored closely for onset of contractions, pulse rate, temperature, bleeding or leaking from the vagina, or other safety issues mentioned below. We monitored the fetal heart rate closely and performed a nonstress test once every 12 h. If not spontaneously expelled, we removed the dilators after 24 h and again noted the Bishop score.

There was no blinding. We started a low-dose oxytocin infusion from 3 mIU/min, incremented by 3 mIU every half hour to a maximum of 24 mIU/min. An artificial rupture of membranes was carried out 4 h after achieving the maximum dose of oxytocin or when the patient started getting three to four contractions in 10 min, each lasting for 30–40 s. After 12 h of artificial rupture of membranes, if the patient failed to achieve active labour (4-cm dilatation with 75%–100% effacement), we considered it as failed induction and delivered by caesarean section. During labour, we monitored the fetal heart pattern electronically with a cardiotocograph (CTG). We carried out emergency caesarean section in case of any intrapartum complications like abnormal fetal heart rate pattern, features of suspected scar dehiscence like scar tenderness, bleeding, or persistent maternal tachycardia. The *primary outcome* was the proportion who delivered vaginally. We also noted the proportion and indications for the emergency caesarean section. The *secondary outcome* was the device's safety, including bleeding, fragmentation, displacement or entrapment, and cervical injury from insertion until removal. We also noted scar dehiscence and maternal or neonatal infections. We used a case record form to note the sociodemographic profile and obstetric details like period of gestation, comorbidities, indication for induction, period of gestation, interpregnancy interval (spacing), and preinduction Bishop score. The intrapartum details noted include post-Dilapan Bishop score, duration of labour, oxytocin duration and maximum dose, any abnormal fetal heart pattern, and mode of delivery. We also noted the previous pregnancy details, complications after device application as mentioned earlier, neonate details, and maternal details till the discharge of the mother and the newborn from the hospital. There were no changes in the trial outcome after the study commenced. We did not require stopping the recruitment as we did not observe any safety issues with the device after enrolling 50% of the sample size.

### 2.5. Statistical Analysis

Continuous variables like age and gestational age were expressed as mean with standard deviation or median with interquartile range (IQR). We expressed categorical variables like parity and maternal and neonatal complications as frequency, percentage, or proportion. We expressed the primary and secondary outcome variables proportionally with a 95% confidence interval (CI). We performed a subgroup analysis of the various factors using the chi-square test to compare proportions and the Student *t*-test to compare means among those who achieved or did not achieve active labour. We performed a multivariable (log binomial) analysis model of all variables with *p* value < 0.2 in the unadjusted analysis. A variable with a *p* value less than 0.05 was considered significant in achieving active labour. The data was collected and analyzed with StataCorp 2015 (Stata: Release 14, Statistical Software, College Station, Texas: StataCorp LP).

## 3. Results

### 3.1. Screening and Recruitment

We screened 172 women. We recruited the participants using systematic random sampling and recruiting every third eligible case attending the first author's unit. However, after March 2020, there was a lockdown due to the COVID-19 pandemic. Since our hospital was also a COVID hospital, the case recruitment got interrupted and converted to consecutive after that. We recruited 82 women, and all of them completed the study. Three of them opted for repeat caesarean after the removal of Dilapan and refused induction of labour with oxytocin.

### 3.2. Outcomes

#### 3.2.1. Labour Outcomes

**Figure 1:**
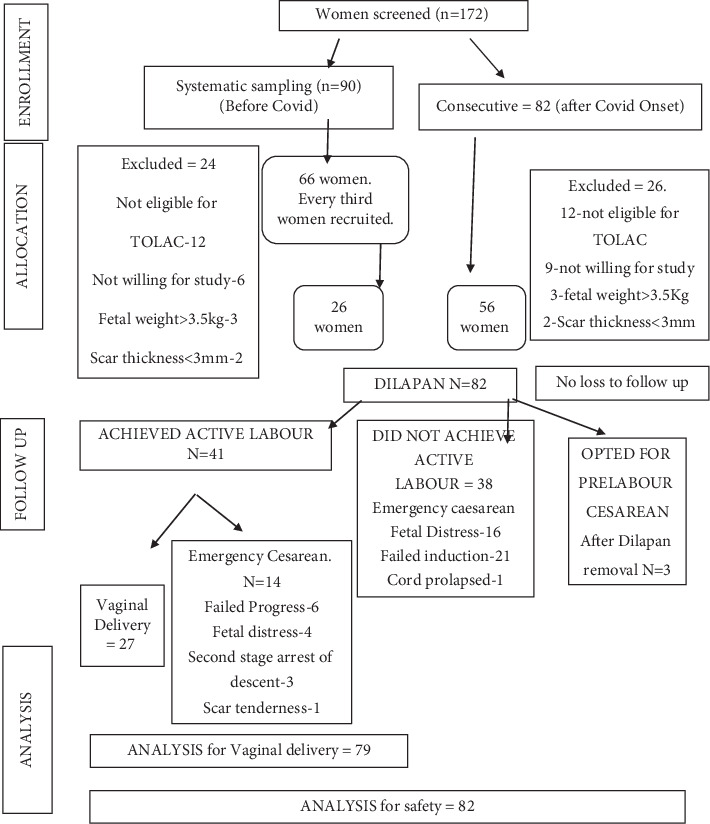
Study flow diagram.

Among the 82 women, three opted for prelabour caesarean section after we removed Dilapan as they did not want to undergo a trial of labour with oxytocin infusion. Seventy-nine women completed the trial of labour. A per-protocol analysis was done. Thirty-eight women did not achieve active labour and required emergency caesarean. The induction successfully achieved an active phase of labour in 41 of the 79 (52%) women. Twenty-seven of the 79 women delivered vaginally, and 52 required emergency caesarean section. Thus, overall, the VBAC rate was 34% (18 spontaneous, nine assisted with forceps, and five with vacuum) (Figure 1).

Table 1 shows the demographic and obstetric details and indication of induction of these 82 participants. Nearly one-fourth of the study population had no comorbidities. Among the others, hyperglycemia and hypertensive disorders were the commonest comorbidities. Fetal distress and failed induction were the most frequent indications for the previous caesarean sections.

Most of the women needed four to five Dilapan. It was inserted for 24 h in 75 (90%) women. The mean duration of insertion was 22.6 h. Two women expelled it spontaneously. The mean Bishop score before the Dilapan insertion was 2.7 (median 3, IQR 2–5), and after the removal of Dilapan, it was 5.3 (median 5, IQR 4–6). The improvement in the Bishop score was statistically significant. The mean increase in the Bishop score was 2.62 (with a CI of −2.96 to −2.287) (*t* = −15.42, *p* < 0.001). The median increase in the Bishop score was also significant (Wilcoxon signed rank test *z* = 7.826, *p* < 0.001). Fifty-eight women required induction of labour, and the rest of the 23 women had spontaneous onset of labour.

#### 3.2.2. Safety

Only one woman had bleeding immediately after insertion. We removed Dilapan immediately, and the bleeding settled. We induced her the next day with oxytocin. She underwent a caesarean for fetal distress. There was no evidence of abruption. None of the women had complications like fragmentation, dislocation entrapment, or injury to the cervix.

Two had scar dehiscence. One woman developed scar tenderness in labour. Emergency caesarean confirmed scar dehiscence. One more had pathologic CTG in the active phase of labour, and we found scar dehiscence during the repeat caesarean section. Both neonates had good Apgar scores at birth. We repaired both the scars. Their recovery was uneventful.

One woman had a traumatic postpartum haemorrhage (PPH) due to vaginal tears after vaginal delivery. The same women had sepsis managed with parenteral antibiotics. She recovered uneventfully.

One woman had antenatally diagnosed fetal demise. She had an uneventful successful vaginal delivery. Other than that, there was no intrapartum stillbirth. Three neonates had an Apgar score less than 7 in the first minute. Four neonates needed admission to the neonatal intensive care unit. Two had respiratory distress at birth, and two had mild jaundice. None of the babies required intubation or ventilation. There was no neonatal mortality.

There was a deviation of protocol in 11 women. We carried out a sweeping stretch of the cervix for nine women after the removal of Dilapan and induction with oxytocin 48 h later. Seven out of these nine had successful vaginal birth. In two women, a Foley catheter was inserted intracervically after removal of Dilapan for another 24 h. Both these women also had a successful vaginal birth.

### 3.3. Factors Associated With Successful Induction

We performed a univariate analysis of different factors between women who achieved (*n* = 41) and those who did not achieve active labour (*n* = 38), as shown in Table 2. On multivariable analysis of the factors as depicted in Table 3, we observed that spontaneous onset of labour after Dilapan removal was associated with a 1.68 times higher chance of reaching the active stage of labour (*p* = 0.027).

## 4. Discussion and Conclusion

We undertook this study to determine the efficacy and safety of the osmotic dilator Dilapan-S for preinduction ripening of the cervix among women with a previous caesarean scar.

Osmotic or hygroscopic dilators are synthetic small rods containing Aquacryl hydrogel. They act by absorbing fluid from the endocervical canal. They swell up and cause dilation of the cervix. They also release prostaglandins, further softening the cervix by breaking down collagen [5]. Clinicians have used it for the termination of pregnancy in the second trimester [8–10]. Authors have recently studied the use of osmotic dilators in unscarred women at term and have found them safe and effective [11–14]. A Cochrane analysis of all these studies showed them safe and effective. Most of these studies have excluded scarred uterus [15]. In the international multicentre study [16] on 543 women, only 41 had previous caesarean. The mean improvement in the Bishop score was 3.6 among them. In the study by the Synthetic Osmotic Cervical Dilator for Induction of Labour in Comparison to Dinoprostone Vaginal insErt (SOLVE) collaboration group [17], only 28 women among the 337 in the Dilapan group had previous caesarean pregnancy.

There are only two published studies on the use of Dilapan-S among women with previous caesarean pregnancy [18]. Maier et al. [19] compared Dilapan-S (*n* = 33) and dinoprostone (*n* = 49) for ripening in 82 women with an earlier caesarean scar. The device was safe and associated with 55% successful VBAC with induction following Dilapan use for ripening. These authors used Dilapan and assessed every 8–12 h. They changed Dilapan and the subsequent new insertion up to three times. They had one scar dehiscence.

In another recently published study [20], the authors found the device safe, with a 52% VBAC rate among 104 women with previous caesarean receiving Dilapan. They compared it with a cohort of 102 women who had received dinoprostone. In this study, the authors removed and reinserted a new dilator up to three times. There were three cases of scar dehiscence in the Dilapan group.

In our study, we found that Dilapan is safe for inducing labour in women with a previous caesarean section eligible for TOLAC. We observed that though 34% achieved VBAC, 52% of the participants progressed to active labour. Other authors [3, 4] have reported a vaginal birth rate of around 50%–60%. We had a lower VBAC rate. The reason could be that we had very few women with previous vaginal delivery. We gave a one-time insertion of Dilapan up to five numbers for 24 h. We did not provide repeat insertions of Dilapan for further ripening if the Bishop score had not improved. We followed a stringent definition for failed induction after starting oxytocin infusion. After 12 h of rupture of membranes, if the women had not achieved an active phase of labour, we performed a caesarean section to terminate the trial. We further observed that spontaneous labour onset after Dilapan insertion increased the odds (1.6 times higher) of achieving active labour. In the study by Saad et al. [21], improved Bishop score and previous vaginal deliveries were predictors of successful vaginal delivery.

The higher VBAC rates among women with a deviation of the protocol in our study suggest that sequential ripening with a Foley single-balloon catheter or sweep stretch of the cervix after Dilapan removal might improve the vaginal birth rates with oxytocin induction.

In conclusion, the osmotic dilator Dilapan-S was effective and significantly improved the Bishop score. There were no incidents of entrapment, upward displacement, fragmentation, or cervical tears. A scar dehiscence was observed in 2.5%. We need more extensive studies to assess scar dehiscence.

Our study adheres to the Transparent Reporting of Evaluations with Nonrandomized Designs (TREND) guidelines (presented as supporting information (available here)).

### 4.1. Limitation

We did not study the satisfaction rates among the women. We did not have a control group. We calculated the sample size assuming a 70% vaginal birth rate. However, we did not achieve that birth rate. We used a clinically assessed Bishop score to assess the cervix. It is less reproducible and subjective [22] and will likely influence the results.

### 4.2. Strengths

The present study is a single-centre study. We included term pregnant women with a prior caesarean section only. We observed strict inclusion and stringent eligibility criteria for TOLAC while recruiting participants. We followed a uniform protocol for induction with oxytocin for all the participants. We monitored all the women with CTG, continuously in labour. There was no case of intrapartum stillbirth.

### 4.3. Research Implications

We suggest randomized trials between single-bulb Foley catheters and hygroscopic dilators among women with a scarred uterus. We also recommend future trials on sequential ripening with a single-balloon Foley catheter after hygroscopic dilators if the Bishop score has not improved to more than 6. We also recommend a more objective method to assess cervical ripening with ultrasonographical measured parameters, especially in research settings [23].

## Figures and Tables

**Table 1:** Clinical–demographic and obstetric profile of the study participants (*N* = 82).

**Parameters**	**N** = 82	
Age (years) (mean ± SD)		28.3 ± 3.7
Indication for induction	Diabetes	19 (23%)
	Hypertension	15 (18%)
	Past dates	30 (37%)
	Oligohydramnios	11 (13%)
	Other	07 (9%)
Previous vaginal delivery	Yes	5 (6%)
	No	77
Previous VBAC	Yes	1 (1.2%)
	No	81
Mean gestational age (weeks) mean ± SD		39 ± 1.2 weeks
Antepartum fetal demise		1
Mean prior Bishop score (mean ± SD)		2.7 ± 1.4
No comorbidities		20 (23.8%)
Interpregnancy interval (months) mean ± SD		48.4 ± 27.1
Scar thickness (mm) mean ± SD		3.5 ± 0.4
Mean duration of Dilapan-S (hours) mean ± SD		22.5 ± 5
Spontaneous expulsion of Dilapan (*n*)		4
Mean (±SD) oxytocin dose (mIU/min)		13.4 ± 8
Mean (±SD) duration of first stage (hours)		11 ± 4.9
Birth weight (mean ± SD) (grams)		2988 ± 488
Rupture of membranes	Spontaneous	24
	Artificial	55
	Caesarean before ROM	3

Abbreviations: ROM, rupture of membranes; SD, standard deviation; VBAC, vaginal birth after caesarean.

**Table 2:** Analysis of variables between groups that achieved and did not achieve active labour.

**Parameter**	**Did not achieve active labour ** **N** = 38	**Achieved active labour ** **N** = 41	**Statistic value**	**p** ** value**
Pre-Bishop score	2.8 ± 1.5	2.7 ± 1.4	*t* = 0.0.17	0.86
Mean ± SD (95% CI)	(2.27–3.24)	(2.15–3.056)		
Indication for previous caesarean section				
Failed induction	6	14		
Labour dystocia	1	0		
Fetal distress	21	17		
Others	10	10	*X* ^2^ = 10.09	0.12
Interpregnancy interval (months)	54.7 ± 31	43.5 ± 22.1	*t* = 1.84	0.07
Mean ± SD (95% CI)	(44.53–64.93)	(36.55–50.47)		
Type of labour				
Spontaneous	3	18	*X* ^2^ = 12.73	0.001⁣^∗^
Induced	33	22		
Oxytocin maximum dose (mIU/min)				
< 10	4	18	*X* ^2^ = 10.93	0.001⁣^∗^
≥ 10	34	23		
Birth weight (grams) mean ± SD	2955 ± 514	3030 ± 457	*t* = −0.69	0.49
Rupture of membranes				
Spontaneous	7	16	*X* ^2^ = 3.23	0.072
Artificial	28	25		
Duration of first stage (hours)				
< 8	4	18	*X* ^2^ = 10.9	0.001⁣^∗^
≥ 8	34	23		
Birth weight (kg)				
< 3	19	20	*X* ^2^ = 0.011	0.91
≥ 3	19	21		

Abbreviations: CI, confidence interval; kg, kilograms; mIU, milli-international units; SD, standard deviation; *t*, Student *t*-test; *X*^2^, chi-square.

⁣^∗^*p* < 0.05 significant.

**Table 3:** Multivariable analysis for factors predicting achievement of active labour.

**Variables**	**Total**	**Active labour**		**Crude risk ratio (95% CI)**	**Adjusted risk ratio (95% CI)**	**p** ** value** ^**a**^
		**n** ** (%)**				
		**Yes**	**No**			
Total	79	41 (51.9)	38 (48.1)	—	—	—
Interpregnancy interval (mean (SD))	79	54.7 (31.0)	43.5 (22.0)	0.99 (0.98–1.00)	0.99 (0.98–1.00)	0.451
Previous caesarean indication						
Failed induction	20	14 (70.0)	6 (30.0)	1.5 (1.02–2.28)	1.38 (0.91–2.11)	0.132
Others	59	27 (45.7)	32 (54.3)	1	1	—
Labour onset (*N* = 76)						
Spontaneous	21	18 (85.7)	3 (14.3)	2.1 (1.48–3.09)	1.68 (1.06–2.67)	0.027⁣^∗^
Induced	55	22 (40.0)	33 (60.0)	1	1	—
Maximum oxytocin dose						
≤ 10 mIU/min	22	18 (81.8)	4 (18.2)	2.0 (1.40–2.94)	1.22 (0.76–1.97)	0.405
> 10 mIU/min	57	23 (40.4)	34 (59.6)	1	1	—
Rupture of membranes (*N* = 76)						
Spontaneous	23	16 (69.6)	7 (30.4)	1.47 (0.99–2.18)	0.91 (0.55–1.52)	0.722
Artificial	53	25 (47.2)	28 (52.8)	1	1	—
Duration of first stage (in hours)						
≤ 8	22	18 (81.8)	4 (18.8)	2.0 (1.39–2.94)	1.59 (1.08–2.36)	0.019⁣^∗^
> 8	57	23 (40.4)	34 (59.6)	1	1	—

Abbreviations: CI, confidence interval; SD, standard deviation.

^a^Variables with *p* value < 0.2 in the unadjusted analysis were included in the multivariable model (log-binomial model).

⁣^∗^*p* < 0.05 significant.

## Data Availability

Data are available on request from the authors.
